# Data on ICP OES and emulsion stability of *Bredemeyera floribunda* root extract: Medicinal plant used by the Brazilian rural population to treat snakebites

**DOI:** 10.1016/j.dib.2019.103940

**Published:** 2019-04-23

**Authors:** Paula Fabiana Saldanha Tschinkel, Elaine Silva de Pádua Melo, Zizelina Mendes Dutra, Nayara Vieira de Lima, Daniela Granja Arakaki, Rafaela Henriques Rosa, Daniel Araujo Gonçalves, Igor Domingos de Souza, Rita de Cassia Avellaneda Guimarães, Danielle Bogo, Valter Aragão do Nascimento

**Affiliations:** a Group of Spectroscopy and Bioinformatics Applied Biodiversity and Health (GEBABS), School of Medicine, Federal University of Mato Grosso do Sul, Campo Grande/MS, Brazil, S/N, Campo Grande, 79070-900, Brazil; b Unidade de Tecnologia de Alimentos e Saúde Pública, Universidade Federal de Mato Grosso do Sul, 79100, Campo Grande, MS, Brazil; c Department of Chemistry, Minas Gerais State University, UEMG, Ituiutaba, Brazil

**Keywords:** *Bredemeyera floribunda*, Traditional medicine, Elemental content, Emulsion stability

## Abstract

*Bredemeyera floribunda* Willd (*B. floribunda*) is a medicinal plant used by the Brazilian rural population to treat snakebites, but there are no data on the elemental composition and stability of the emulsion of extract of this plant. In this article, we present for the first time data on contents obtained the roots of the medicinal plant *B. floribunda* by using inductively coupled plasma optical spectrometry (ICP OES), as well as the data on preliminary stability tests of emulsions formulated with aqueous extract of this plant. The first set of data shows a total of 13 elements (Al, Ca, Cr, Fe, K, Mg, Mn, Na, Ni, P, Cu, Se and Zn) detected in the roots of the plant. The second set of data shows that the extract data of the *B. floribunda* roots presented an emulsifying potential. In addition, the article provides information on the heating program for microwave oven digestion and ICP OES operating conditions. The data presented make way for nutritional and toxicological studies involving the roots of *B. floribunda*. The data on the cataloging of the plant can be found in the deposit nº 54366 of the Herbarium of the Federal University of Mato Grosso do Sul, available in www.splink.org.br/form?lang=pt&collectioncode=CGMS*.*

**Table:** Specifications Table

Subject area	Biochemistry
More specific subject area	Plant medicine
Type of data	Table
How data was acquired	Microwave oven (Speedwave four, Berghof, Eningen, BW, Germany) and ICP OES (iCAP 6300 Duo, Thermo Fisher Scientific, Bremen, Germany) Drying oven (Lawes 880X52T); Thermostatic Bath (FISATOM series, 034365 model: 550); pH meter (HANNA, Model pH 21).
Data format	Raw roots, Analyzed
Experimental factors	Pretreatment methods for the digestion of samples: (i) The roots underwent a process of drying (samples were drying for 24 h in a hot oven at 40°C); (ii) The dried roots were triturated using the Thermomix TM5 equipment (Vorwerk LLC, USA); (iii) In 0.25 g of triturated roots was added 3 ml of HNO_3_ (65% Merck), 1 ml of H_2_O_2_ (35%, Merck) and 2.0 ml of high-purity water; Preparation of the aqueous extract of the *B. floribunda* root. (i) Roots were washed with high-purity water, and later ground in a knife mill; (ii) Subsequently the crushed roots were oven dried at 40 °C for 48 hours; (iii) The aqueous extract was obtained by the hot maceration technique, subsequently the material is left in contact with the extracting solvent (distilled water) for 30 minutes;
Experimental features	Determination of concentration levels of contents (Al, Ca, Cr, Fe, K, Mg, Mn, Na, Ni, P, Cu, Se and Zn) in the root of the medicinal plant *B. floribunda*. Development and evaluated the physical stability of emulsions formulated with aqueous extract of the *B. floribunda* root.
Data source location	Bebedouro settlement, Nova Alvorada do Sul, Mato Grosso do Sul, Brazil, W -21.467389; S -54.383361, identified by Dr. Flávio Macedo Alves and Geraldo Damasceno Júnior
Data accessibility Related research article	Deposited in the herbarium of Federal University of Mato Grosso do Sul under the number CGMS 54366 available in: www.splink.org.br/form?lang=pt&collectioncode=CGMS Rocha et al. [1]. Data on elemental composition of the medicinal plant *Hymenaea martiana* Hayne (Jatobá), Data in Brief, 19(2018), pp. 959–964.doi: 10.1016/j.dib.2018.05.142

**Table:** 

**Value of the data** • The elemental quantification is useful in preventing accidental toxicity in use of *B. floribunda* roots as a medicine. • The data obtained of elemental contents of *B. floribunda* can be compared with other results published in the literature on medicinal plants. • The extract data of the *B. floribunda* roots presented an emulsifying potential and can be inserted in the alimentary area, providing a new alternative to the allergic individuals to certain already commercialized emulsifiers. • This data may be useful for the pharmaceutical industry and assist in the development of new herbal medicines.

## 1 Data

The data presented in subsection 1.1 include results on elemental content in *Bredemeyera floribunda* Willd (Polygalaceae) roots detected by inductively coupled plasma optical atomic emission spectrometer (ICP OES) referred in Table 1, presented in mg/Kg according to international standardization.

**Table 1:** Analytical data on elemental content present in the *B. floribunda* roots detected in ICP OES (in units of mg/Kg ± standard deviation of triplicate).

Elements	*B. floribunda* mg/Kg
Al	238.30 ± 7.80
Ca	2843.9 ± 92.12
Co	< LOD
Cr	2.70 ± 0.04
Cu	18.79 ± 1.86
Fe	387.07 ± 9.50
K	551.50 ± 16.80
Mg	318.40 ± 9.24
Mn	20.80 ± 0.50
Na	177.75 ± 6.40
Ni	0.80 ± 0.02
P	546.60 ± 11.50
Se	458.90 ± 15.85
Zn	8.13 ± 0.16

< LOD - Analyte concentrations were below the limits of detection.

In subsection 1.2 we presented data on the characterization studies performed with the measurement of pH, and de-emulsification index for 240 minutes, centrifugations test at 1000, 2500 and 3500 rpm and thermal stress from 40 to 80 °C (Table 2). The latter data (subsection 1.3, Table 3) includes the Freeze-thaw cycle in a total of 6 cycles, each one consisting by the cooling the samples to 4 °C for 24 hand then heating it to 45 °C for 24 h; initial pH, final pH and accelerate stability test for 30 days.

**Table 2:** Data obtained from de-emulsification index tests, centrifugations test, thermal stress and pH analysis of the formulations containing the *B. floribunda* extract and their respective standards.

Avaliations		E1	E2	E3		P1		P2	P3
De-emulsification index (ml)									
0 min.	0		0		0		0	0	0
15 min.	0		0		0		0	0	0
30 min.	0		0		0		0	0	0
60 min.	0		0		1.36 ml		0	0	0
120 min.	1.91 ml		0		3.36 ml		0	0	1.63 ml
240 min.	3.9 ml		0		5.4 ml		0	0	2.7 ml
Total de-emulsification	5.81 ml		0		10.12 ml		0	0	4.33 ml
Centrifugations test	Stable		Stable		Partly stable		Stable	Stable	Partly Stable
Thermal stress (Temperature)	N (40–80 °C)		N (40–80 °C)		PD (40–80 °C)		N (40–80 °C)	N (40–80 °C)	PD (40–80 °C)
Final pH^1)^	3.6		4.0		3.42		–	8.45	8.76

E1 = pure extract, E2 = extract and sugar; E3 = extract and sodium chloride; P1 = standard (egg white); P2 = standard (egg white) and sugar, P3 = standard (egg white) and sodium chloride; Final pH^1)^ = thermal stress; PD = partial destabilization; N = normal (there were no changes).

**Table 3:** Data obtained from Freeze-thaw cycle tests, accelerated stability tests and pH analysis of the formulations containing the *B. floribunda* extract and their respective standards.

Avaliations	E1	E2	E3	P2	P3
Freeze-thaw cycle (ml)					
1º cycle	<1 ml	0	<1 ml	0	<1 ml
2º cycle	3 ml	<1 ml	3.58 ml	1.46 ml	2.33 ml
3º cycle	5.06 ml	2.50 ml	5.70 ml	3.33 ml	5.53 ml
4º cycle	8.33 ml	3.66 ml	10.46 ml	5.16 ml	8.23 ml
5º cycle	6.40 ml	6.66 ml	DT	8.33 ml	DT
6º cycle	DT	7.23 ml	–	10.51 ml	–
Initial pH^2)^	3.8	4.03	3.44	8.52	8.81
Final pH^3)^	4.0	4.3	3.5	8.0	8.5
Accelerated stability tests					
3rd day (ml)	<1.0	<1.0	5.4	<1.0	4.5 ml
7th day (ml)	3.0	2.5	DT	3.0	DT
15th day (ml)	4.5	4.25	DT	4.5	DT
30th day (ml)	4.5	4.25	DT	4.5	DT
Initial pH^4)^	3.8	4.03	3.44	8.52	8.81
Final pH^5)^	3.4	5.2	3.0	8.64	7.4

E1 = pure extract, E2 = extract and sugar; E3 = extract and sodium chloride; P2 = standard (egg white) and sugar, P3 = standard (egg white) and sodium chloride; Initial pH^2)^ = Freeze-thaw cycle, Final pH^3)^ = Freeze-thaw cycle; DT = de-emulsification; initial pH^4)^ = accelerated stability testing, final pH^5)^ = accelerated stability testing.

### 1.1 Data analysis by ICP OES

In Table 1, a total of 13 elements (Al, Ca, Cr, Fe, K, Mg, Mn, Na, Ni, P, Cu, Se and Zn) were quantified in the roots of the *B. floribunda*. The data in Table 1 show that Co level was below limit of detection in root samples. The experiments were analyzed by ICP OES after digestion procedures (Table 4, Table 5).

**Table 4:** Operating program for the microwave digestion system.

Step	Power (%)^a^	Temperature (°C)	Time (min)	Pressure (Bar)
1	90	160	5	35
2	90	190	10	35
3	0	50	10	0

a 100% power correspond to 1450 W.

### 1.2 Data on physical stability obtained from macroscopic analysis, de-emulsification index tests, centrifugations test, thermal stress and pH analysis

#### 1.2.1 Macroscopic analysis and emulsifying capacity

Macroscopic analysis of the tested formulations revealed that the aqueous extracts of the plant roots had a creamy, white, shiny appearance. Analysis of the emulsion type determination test and study of the emulsifying capacity revealed that the emulsion presented an aqueous external phase, characterized as O/A (oil/water) type emulsion. However, such extract may be used in O/A or A/O (water/oil) formulations, since the amount of the dispersed (oily) phase should be high in volume to destabilize the emulsion. In carrying out the emulsion type test and emulsifying ability study, it was found that 680 ml of oil is required to totally destabilize the emulsion. On the other hand, an amount of 560 ml of oil is required to initiate the disestablishment of 35 ml of the aqueous extract. The pure extract (E1) presented superior characteristics when compared to standard (egg white (P1)) in the expansion versus volume aspect. By means of this test, it was possible to analyze that 10 mL of emulsion of the pure extract with sugar (E2) (1: 1 v/v) presented expansion volume of 150 mL, whereas 10 mL of the standard (egg white (P1)) with sugar (1: 1 v/v) presented the final volume of 30 mL.

#### 1.2.2 De-emulsification index tests

Data on physical stability obtained from de-emulsification index tests, centrifugations test, thermal stress and pH analysis of the formulations containing the *B. floribunda* extract and their respective standards are presented in Table 2. For 30 minutes after the emulsions were elaborated, no sample had de-emulsification. After 60 minutes the E3 (extract and sodium chloride) sample showed initial de-emulsification. Samples P3 (standard (egg white) and sodium chloride) and E1 showed initial de-emulsification after 120 minutes of emulsion preparation. On the other hand, E3 after 120 minutes presented intermediate de-emulsification index. At the end of the 240 minutes, only samples P2 (standard (egg white) and sugar) and E2 did not present de-emulsification, E3 and P3 presented total de-emulsification, and sample E1 presented an intermediate de-emulsification index.

#### 1.2.3 Centrifugations test

The formulations E1, E2, P1 and P2 were approved in the centrifugation test; however the formulation with the plant extract showed higher resistance to centrifugation compared to the standard.

#### 1.2.4 Thermal stres

Samples with sodium chloride addition (E3 and P3) did not withstand the process, i.e. the formulations were intensively modified after the centrifugation test (Table 2). Samples P3 and E3 are partially stable according to the temperature rise from 40 °C to 80 °C, in fact, the process interfered in the stability of the samples, which released water, occurring phase separation. It is observed in Table 2 that some samples (E1, E2, P1 and P2) submitted to thermal stress were stable and no phase separation occurred.

### 1.3 Data on physical stability obtained from freeze-thaw cycle tests, accelerated stability tests and pH analysis

#### 1.3.1 Freeze-thaw cycle tests

The data in Table 3 show the results obtained from Freeze-thaw cycle tests, accelerated stability tests and pH analysis of the formulations containing the *B. floribunda* extract and their respective standards. As shown in Table 3, only the E2 and P2 samples were stable at the end of the Freeze-thaw cycle tests. The E2 sample had a lower de-emulsification index compared to P2 (Table 3). There was a variation of the pH of the samples, however, occurring with higher intensity in the samples with egg white.

#### 1.3.2 Accelerated stability tests

The results of the accelerated stability study revealed that on the 3rd day only the E3 and P3 samples had de-emulsification index >1 mL (E3 = 5.4 mL and P3 = 4.5 mL). In addition, on day 7 samples E3 and P3 had a total de-emulsification index and were discarded.

According to the data in Table 3, samples E1, E2 and P2 remained stable after the end of 30 days. The E2 sample has the lowest de-emulsification rate within 30 days (4.25 mL). However, samples E1 and P2 reached the same de-emulsification index (4.5 mL) over the course of 30 days. After the 15th day, there was no de-emulsification of samples E1, E2 and P2, which is interesting from the commercial point of view, since the functional characteristics of a final product can influence its shelf life without any retrogradation or release of liquids from the product over time.

## 2 Experimental design, materials and methods

### 2.1 Plant material

*Bredemeyera floribunda* Willd was collected in June 2016 from (rural region) in the Nova Alvorada region/Brazil (Coordinates: 21°46'73.89"S; 54°38'33.61"W. Altitude 510 m). The specimen (deposit number 54366 CGMS) was deposited on June 4th^,^ 2017 at Biology Department of Biology Herbarium (UFMS). The plant was identified by biologists Flávio Macedo Alves and Geraldo Alves Damasceno Junior. The project was registered in the National System of Genetic Resource Management and Associated Traditional Knowledge (SisGen, nº A7716EC).

### 2.2 Microwave-assisted digestion

The roots were subjected to a drying process for 24 h in a hot oven at 40 °C. Subsequently, the dried samples were crushed and sieved (stainless steel sieve, 200 μm granulometry). Approximately an amount of 0.25 g roots of *B. floribunda* was digested with 3.0 mL of HNO_3_ (65%, Merck), 1.0 mL of high-purity water (18 MΩ cm, Milli-Q, Millipore, Bedford, MA, USA) and 2.0 mL of H_2_O_2_ (35%, Merck) in microwave digestion system (Speedwave four, Berghof, Eningen, BW, Germany), according to the digestion program presented in Table 4 [1]. The resulting solutions were cooled and diluted to 30 mL with high-purity water. A similar digestion was performed for standard reference material, trace elements in Pine Needles (SRM 1575a), and certificate by National institute of standards and technology - NIST.

After digestion step, the samples were analyzed by ICP OES. High purity argon 99,996% (White-Martins-Praxair) was utilized to purge the optic, plasma generation.

### 2.3 Process of data analysis by ICP OES

The contents of macro and microelements (Al, Ca, Co, Cr, Cu, Fe, K, Mg, Mn, Na, Ni, P, Se and Zn) in *B. floribunda* were determined by ICP OES (iCAP 6300 Duo, Thermo Fisher Scientific, Bremen, Germany). The calibration curves and stock solution to mineral detection were set up with high-purity water and nitric acid. The concentrations of the different elements in these samples were measured using the corresponding multi elementary standard solution (100 mg L^−1^) containing Al, Ca, Co, Cr, Cu, Fe, K, Mg, Mn, Na, Ni, P, Se and Zn (Specsol, São Paulo, Brazil). Axial view was used for elements determination and 3 replicates were used to measure the analytical signal. All glassware were soaked in nitric acid (10% v v^−1^) during 24 h for decontamination. ICP OES operating conditions are summarized in Table 5.

**Table 5:** Operating conditions for ICP OES analysis.

Parameters	Setting
RF Power (W)	1250
Sample flow (L min^−1^)	0.45
Replicates	3
Plasma flow rate (L min^−1^)	12
Integration time (s)	15
Stabilization time (s)	20
Nebulization pressure(psi)	20
Plasma View	Axial
Analytes/Wavelength	Al 396.100 nm; Ca 422.673 nm;
	Co 228.616 nm; Cr 267.716 nm;
	Cu 324.754 nm; Fe 259.940 nm;
	K 766.490 nm; Mg 279.553 nm;
	Mn 257.610 nm; Na 588.995 nm;
	Ni 221.647 nm; P 214.914 nm;
	Se 196.09 nm; Zn 213.856 nm;

Table 6 show limit of detection (LODs) calculated according to IUPAC as 3 times standard deviation from blank sign (SB) divided by calibration curve slope (m): LOD = 3*SB/m [2]. The limit of quantification (LOQs) were calculated in the same way, but, LOQ = 10*SB/m. The correlation coefficient (*R*^*2*^) obtained from the calibration curves reached values of 0.999 for all analytes.

**Table 6:** Analytical characteristics of ICP OES method: Limit of detection (LODs), limit of quantification (LOQs) and correlation coefficient (R^2^*)*.

Elements	LOD (mg L^−1^)	LOQ (mg L^−1^)	*R*^*2*^
Al	0.020	0.006	0.9998
Ca	0.010	0.050	0.9990
Co	0.002	0.008	0.9998
Cr	0.010	0.040	0.9998
Cu	0.002	0.005	0.9998
Fe	0.002	0.006	0.9998
K	0.002	0.006	0.9999
Mg	0.009	0.030	0.9990
Mn	0.002	0.005	0.9999
Na	0.040	0.100	0.9999
Ni	0.002	0.007	0.9999
P	0.020	0.060	0.9997
Se	0.001	0.003	0.9993
Zn	0.001	0.003	0.9990

The trueness of data obtained by the proposed method, using standard Reference Material - SRM 1575a (Pine Needles) is presented in Table 7. According to the t-test, there was no statistical difference between the values of the contents quantified by ICP OES and stipulated by the reference material. The use of certified reference materials is an important pillar for the assessment of the quality of any acquired analytical data.

**Table 7:** Comparison of results obtained by ICP OES with values of the reference certified material (SRM).

Elements	ICP OES	SRM 1575a	t-test^a^
Al	550.16 ± 30.00	580.00 ± 30.00	1.4334
Ca	2090.80 ± 290.69	2500.0 ± 100.0	2.3054
Cd	0.230 ± 0.006	0.230 ± 0.004	0.1227
Cd	0.054 ± 0.014	0.061 ± 0.002	0.8040
Cr	0.440 ± 0.130	0.3–0.5^b^	
Cu	2.89 ± 0.31	2.8 ± 0.2	0.4517
Fe	42.17 ± 2.90	46.0 ± 2.0	1.8839
K	3530.48 ± 364.76	4170.0 ± 70.0	2.9822
Mg	872.90 ± 15.41	1060.0 ± 160.0	2.0160
Mn	422.62 ± 50.0	488.0 ± 12.0	2.7764
Na	64.63 ± 4.47	63.0 ± 1.0	0.6161
Ni	1.07 ± 0.27	1.47 ± 0.10	2.3798
P	1018.06 ± 2.04	1070.0 ± 80.0	1.1241
Zn	36.0 ± 0.14	38.0 ± 2.0	1.7505

a Critical t = 4.3026.

b Mass fraction information values (dry base) SRM 1575a Concentration expressed in mg/Kg ± standard deviation, n = 3.

### 2.4 Preparation of the aqueous extract of the B. floribunda root

The roots collected from the plant were sanitized and ground in a knife mill, and then placed in a drying oven, ventilated, thermostatically controlled, operating at 40 °C for 48 hours. The aqueous extract was obtained by the hot maceration technique, in which the material is left in contact with the extracting solvent (distilled water) for 30 minutes in a thermostatic bath (FISATOM series 034365 mod. 550) at 65 °C or until the extract reaches the Soluble solids content of 0.3° BRIX. After this time, the solution was gauze filtered. The volume of extracting solvent used was 800 ml for each 200 g of material for extraction.

The egg whites were used as the comparative standard. The following formulations were performed and tested: E1 pure extract, E2 extract and sugar (10 g), E3 extract and sodium chloride (1 g), P1 standard (egg white), P2 standard (egg white) and sugar (10 g), P3 standard (egg white) and sodium chloride (1 g).

### 2.5 Macroscopic analysis of the formulations

The macroscopic observation of the formulations was performed after 24 hours of sample preparation. It was observed the organoleptic characteristics and the homogeneity of the formulations in order to identify the probable process of instability such as cream, flocculation, coalescence and principally de-emulsification index [3].

### 2.6 Determination of extracts pH

The pH values of the formulations were determined directly in the samples just after preparation of emulsions using a pH meter (HANNA, Model pH 21) at 25 ± 1 °C. All experiments were replicated three times.

### 2.7 Determination of the type of emulsion and study of the emulsifying capacity

Experimental part performed in order to identify the oil-in-water (O/W) or water-in-oil (W/O) types of emulsion [4]. The emulsifying capacity of formulations was determined considering the volume (ml) of oil added until reaching the point of inversion of the emulsion using 35 ml of the aqueous extract.

### 2.8 Preliminary stability tests

Preliminary stability tests were replicated five times. The emulsions classified macroscopically as stable after 24 hours of manipulation were subjected to the following preliminary stability tests:

• Centrifugation test**:** For the centrifugation test, 25 mL formulations were submitted to three different rotation speeds: 1000, 2500 and 3500 rpm for 15 minutes at room temperature (FANEM EXCELSA II mod. 206 BL) [5].
• Thermal stress test: This methodology was adapted from Ref. [5]. The test emulsions were conditioned in polystyrene plastic bottles and subjected to heating in a thermostated bath in the temperature range of 40–80 °C (FISATOM series 034365 models 550). Every 30 minutes there was temperature increase of 10 ± 1 °C. Analyses were taken after the sample reached 80 °C, and after the samples were cooled to room temperature. To verify possible changes in the stability of the emulsions, the samples that remained macroscopically stable were evaluated from the following parameters: macroscopic analysis and determination of the pH value.
• Freezing and thawing cycle test was applied for investigate our samples [6], [7]. The formulations were placed in a refrigerator at 4 ± 2 °C for 24 hand then in an oven at 45 ± 2 °C for 24 hours (Refrigerator duplex, Model CRM51AK. Consul), thereby completing one cycle. The first day corresponds to 24 hours after preparation of the formulations. The analyses were performed before the start of the test and at the end of the 6th cycle (12 days). To verify the possible changes in emulsion stability, macroscopic analysis and determination of pH were used.
• Accelerated stability test: The formulations were conditioned in polystyrene plastic bottles and then subjected to the following temperature conditions: 1st day at 4 ± 2 °C (Refrigerator duplex, Model CRM51AK. Consul), 2nd day at 25 ± 2 °C (Controlled Environment Temperature), 3rd day at 37 ± 2 °C and 4th day at 45 ± 2 °C. Analyzes were performed before the start of the test (24 hours after the preparation of the formulations) and at the 7th, 15th, and 30th days. The parameters evaluated were visual macroscopic characteristics and pH value.
