# Supplementary material for: Data on ICP OES and emulsion stability of Bredemeyera floribunda root extract: Medicinal plant used by the Brazilian rural population to treat snakebites
Source: Data Brief. 2019 Apr 23;24:103940. doi: 10.1016/j.dib.2019.103940 (PMC6503127; doi:10.1016/j.dib.2019.103940)
Supplement: Multimedia component 1 [file mmc1.pdf]

Conflicts of Interest Statement

Manuscript title: **Data on ICP OES and emulsion stability of *Bredemeyera floribunda* root extract: Medicinal plant used by the Brazilian rural population to treat snakebites.**

The authors whose names are listed immediately below certify that they have NO affiliations with or involvement in any organization or entity with any financial interest (such as honoraria; educational grants; participation in speakers' bureaus; membership, employment, consultancies, stock ownership, or other equity interest; and expert testimony or patent-licensing arrangements), or non-financial interest (such as personal or professional relationships, affiliations, knowledge or beliefs) in the subject matter or materials discussed in this manuscript.

Author names:

Paula Fabiana Saldanha Tschinkel Paula F. S. Tschinkel 27/02/2019

Elaine Silva de Pádua Melo Elaine S. de Pádua Melo 27/02/2019

Zizelina Mendes Dutra Zizelina Mendes Dutra 27/02/2019

Nayara Vieira de Lima Nayara Vieira de Lima 27/02/2019

Daniela Granja Arakaki Daniela Arakaki 27/02/2019

Rafaela Henriques Rosa Rafaela Rosa 27/02/2019

Daniel Araujo Gonçalves [illegible]

Igor Domingos de Souza Igor Domingos de Souza 27/02/2019

Rita de Cassia Avelhaneda Guimarães Rita de Cássia A. Guimarães 27/02/2019

Danielle Bogo Danielle Bogo 27/02/2019

Valter Aragão do Nascimento Valter Aragão 27/02/2019
